# Mpp10 represents a platform for the interaction of multiple factors within the 90S pre-ribosome

**DOI:** 10.1371/journal.pone.0183272

**Published:** 2017-08-16

**Authors:** Bebiana Sá-Moura, Markus Kornprobst, Satyavati Kharde, Yasar Luqman Ahmed, Gunter Stier, Ruth Kunze, Irmgard Sinning, Ed Hurt

**Affiliations:** Biochemistry Center Heidelberg BZH, University of Heidelberg, Heidelberg, Germany; University of Edinburgh, UNITED KINGDOM

## Abstract

In eukaryotes, ribosome assembly is a highly complex process that involves more than 200 assembly factors that ensure the folding, modification and processing of the different rRNA species as well as the timely association of ribosomal proteins. One of these factors, Mpp10 associates with Imp3 and Imp4 to form a complex that is essential for the normal production of the 18S rRNA. Here we report the crystal structure of a complex between Imp4 and a short helical element of Mpp10 to a resolution of 1.88 Å. Furthermore, we extend the interaction network of Mpp10 and characterize two novel interactions. Mpp10 is able to bind the ribosome biogenesis factor Utp3/Sas10 through two conserved motifs in its N-terminal region. In addition, Mpp10 interacts with the ribosomal protein S5/uS7 using a short stretch within an acidic loop region. Thus, our findings reveal that Mpp10 provides a platform for the simultaneous interaction with multiple proteins in the 90S pre-ribosome.

## Introduction

Ribosomes are ancient molecular machines essential for cell growth and viability. Eukaryotic ribosome assembly is a complex process that extends from the nucleolus to the cytoplasm and requires the assembly of 4 different ribosomal RNAs (rRNAs) with 79 ribosomal proteins (r-proteins). It is a highly coordinated and ordered multistep process that starts in the nucleolus with transcription of a long polycistronic transcript (called 35S pre-rRNA in yeast) by RNA polymerase I. This precursor carries the respective RNAs from both the 40S (18S rRNA) and the 60S subunits (5.8S and 25S rRNA) that are separated by the internal transcribed spacers 1 and 2 (ITS1 and ITS2), and flanked by the 5′ and 3′ external transcribed spacers (5′-ETS and 3′-ETS). These spacer sequences are removed during ribosome assembly. RNA polymerase III is responsible for the transcription of the 5S rRNA precursor that will become part of the large ribosomal subunit [1]. Ribosome biogenesis factors, early binding r-proteins and small nucleolar ribonucleoprotein particles (snoRNPs) associate co-transcriptionally with the nascent 35S pre-rRNA originating a huge macromolecular complex, termed 90S pre-ribosome or small-subunit processome [2, 3]. The 90S particle is composed of approximately 70 assembly factors that form stable sub-complexes, including the UTP-A/tUTP, UTP-B, UTP-C, Mpp10-Imp3-Imp4, Bms1-Rcl1, and U3 snoRNP modules, which associate in a sequential and hierarchical manner with the nascent pre-rRNA [4–6]. Within the 90S particle a series of initial cleavages occur in the 5’-ETS of the rRNA at sites A0, A1 followed by cleavage in ITS1 at site A2, yielding the 20S (precursor to 18S) and 27S pre-rRNAs (precursor to 5.8S and 25S), respectively, thereby separating the maturation pathways of the small and large subunit.

UTP-A and UTP-B are multi-protein complexes, which consist predominantly of WD40 (β-propeller) and α-helical domains that have been suggested to possess a structural role in early ribosome biogenesis steps. Recent cryo-EM structures of the 90S particle provided a first structural view into its molecular architecture, revealing that UTP-A and UTP-B form a mold-like scaffold in which the nascent 18S pre-rRNA is folded, modified and processed [7–9]. The U3 snoRNP, composed of the U3 snoRNA, the box C/D proteins and U3-specific factor Rrp9, is essential for both correct folding of the pre-rRNA as well as processing events at sites A0, A1 and A2 [10–15]. U3 snoRNP is strategically positioned at the center of the particle, allowing the formation of heteroduplexes between the U3 snoRNA and the 5’-ETS and several regions within the 18S pre-RNA [7, 10, 13, 16]. Base pairing between the U3 snoRNA and the 18S rRNA has been suggested to be involved in preventing premature formation of the central pseudoknot, an important architectural feature at the center of the mature 40S subunit [11].

The Mpp10 complex, consisting of the long disordered protein Mpp10, the uS4-like factor Imp3, and the Brix-domain protein Imp4 [17] was identified at the center of the 90S pre-ribosome adjacent to the UTP-B module and close to the 5’ region of the U3 snoRNA [7–9]. Mpp10, and its bona fide binding partners Imp3 and Imp4 have been shown to be involved in chaperoning U3::pre-18S rRNA hybrid formation by destabilization of the intramolecular box A/A’ helix of the U3 snoRNA and stabilization of the U3-hybridized state [18].

In this study, we sought to further investigate the role of the Mpp10 complex in ribosome biogenesis. We further extended Mpp10’s factor network by identifying two new binding partners, the biogenesis factor Utp3/Sas10 and the ribosomal protein Rps5/uS7 and mapped the respective regions within Mpp10 required for these interactions. Furthermore, we present the crystal structure of the Imp4-Mpp10 complex from the thermophilic fungus *Chaetomium thermophilum* at 1.88 Å resolution and analyze it in the context of previously published cryo-EM density of the 90S particle.

## Material and methods

### Yeast strains, plasmids and plasmid constructions

Complete lists of the plasmids and *Saccharomyces cerevisiae* strains used in this study are presented in S1 and S2 Tables, respectively. Yeast manipulations were carried out according to standard procedures [19]. Gene disruption and C-terminal tagging were performed as previously described [20, 21].

### Yeast-2-hybrid assay

Yeast-2-hybrid analysis was carried out as previously described [22]. The *C*. *thermophilum* ORFs and truncations were cloned into appropriate vectors that allow expression of these proteins as N-terminal fusion proteins carrying either the transcription-activating domain (AD) or DNA-binding domain (BD) of the yeast Gal4 transcription factor. Both plasmids (LEU2 and TRP1 marker, respectively) were co-transformed into the haploid reporter yeast strain PJ69-4, which allows detection of weak (HIS3 reporter) and strong interactions (ADE2 reporter). Colonies were spotted onto SDC-Leu-Trp (spotting control), SDC-Trp-Leu-His (weak interactions) and SDC-Trp-Leu-Ade plates (strong interactions), respectively, and grown for 3–4 days at 30°C. The Y2H vector combination SV40 (AD) and p53 (BD) served as positive interaction control and also to detect potential self-activation of the investigated construct.

### Tandem affinity purification from *S*. *cerevisiae* and *C*. *thermophilum*

Tandem affinity purification of FTpA-tagged (Flag-TEV-proteinA) yeast bait proteins or split-tag tandem-affinity purification of co-expressed *C*. *thermophilum* proteins in yeast, were performed in a TAP buffer containing 50 mM Tris-HCl (pH 7.5), 150 mM NaCl, 1.5 mM MgCl2, 5% v/v glycerol, 0.1% v/v NP-40, and 1 mM DTT as previously described [23, 24]. Pellets were thawed and resuspended in ~25 ml TAP buffer supplemented with protease inhibitor mix FY (Serva). Lysis of yeast cells was carried out by adding ~25 ml cold glass beads to the cell suspension and using a bead beater (Pulverisette, Fritsch, Germany). The lysate was first pre-cleared from the glass beads and then centrifuged (Beckman Coulter centrifuges, rotor JA-25.50, 17,000 rpm for 20 min at 4°C). The resulting supernatant was used for tandem affinity purification. In the first step, the supernatant was incubated with ~300μl slurry of pre-equilibrated IgG Sepharose beads (IgG SepharoseTM Fast Flow, Amersham Bioscience) for at least ~2 hours on a turning wheel at 4°C. After binding, IgG beads were once batch-washed in TAP buffer and then with another 15 ml (without protease inhibitors) using a Mobicol minispin column (MoBiTec, Germany). For TEV protease cleavage, TAP buffer (containing 1 mM DTT) supplemented with an aliquot of self-made recombinant HIS-TEV protease (from a 1–5 mg/ml -80°C frozen stock solution) was added to the IgG beads and incubated for another 90 min on a turning wheel at 16°C. The collected TEV eluate was transferred to a new Mobicol minispin column, containing ~50μl slurry of pre-equilibrated Flag M2 agarose beads (anti-FLAG M2 Affinity Gel, Sigma-Aldrich) and binding was performed for at least 1 hour on a turning wheel at 4°C. Flag beads were washed and co-precipitating proteins/particles were eluted by adding TAP buffer supplemented with 1.5X Flag peptide (Sigma-Aldrich).

### Protein coexpression in *E*. *coli* and *in vitro* binding assays

Frozen pellets of *E*. *coli* cells were slowly thawed and resuspended in ~25 ml of binding buffer containing 20 mM HEPES (pH 7.5), 150 mM NaCl, 5% v/v glycerol, 1 mM DTT and 0.01% v/v NP-40 (IGEPAL CA-630) and lysed using a microfluidizer (Microfluidics Corp., MA, USA). The lysate was cleared by centrifugation (Beckman Coulter centrifuges, rotor JA-25.50, 17,000 rpm for 20 min at 4°C) and the supernatant was used for binding assays. For purification of GST-tagged proteins (glutathione-S-transferase tag), ~300 μl slurry of pre-equilibrated Glutathione-Sepharose (Sigma) beads was added and incubated at 4°C for 1 hour and 30 min on a turning wheel. After a washing step of the beads using a Mobicol minispin column (MoBiTec, Germany), GST-tagged proteins and the bound material were eluted by adding binding buffer supplemented with 20 mM glutathione (GSH) to the beads and incubating on a turning wheel for 20 min at room temperature. Samples were analyzed by SDS-PAGE and Coomassie staining.

### Protein expression and purification for crystallization

Untagged *ct*Imp4 (residues 76–274) and His_6_-ZZ-*ct*Mpp10 (residues 433–562) were expressed separately in Rosetta 2 pLysS cells in ZYM5052 auto-induction media [25]. Cells were incubated at 37°C until OD_600_ reached 0.8–1.0 and then shifted to 22°C for 16–18 hours. Cells were harvested by centrifugation for 20 minutes at 4000 rpm, resuspended in lysis buffer (20 mM Na-HEPES pH 7.5, 250 mM NaCl, 40 mM Imidazole, 5 mM MgCl_2_) and co-lysed with a Microfluidics Fluidizer M110L (Microfluidics Corp., MA, USA). Cell debris and insoluble material were removed by centrifugation at 30000× g for 30 minutes (JA-25.50 rotor). The supernatant was applied to a 1–2 mL NiNTA column and washed with lysis buffer. The complex was eluted with lysis buffer supplemented with 250 mM Imidazole. The His_6_-ZZ tag on *ct*Mpp10 was cleaved with His_6_-TEV protease and simultaneously dialysed against SEC buffer (20 mM HEPES pH 7.5, 150 mM NaCl, 5 mM MgCl_2_, 1% glycerol). His_6_-TEV and His_6_-ZZ were removed by reverse NiNTA chromatography. The flow-through containing *ct*Imp4-*ct*Mp10 complex was concentrated and used for size exclusion chromatography using a Superdex 75 26/60 equilibrated with SEC buffer. Fractions containing pure *ct*Imp4-*ct*Mpp10 complex were concentrated to 9–16 mg/mL and used for crystallization trials.

### Crystallization and structure determination

Needle shaped crystals of *ct*Imp4-*ct*Mpp10 complex grew in conditions containing 25–30% ethylene glycol and were harvested directly without further cryo-protection. Diffraction data were collected from a single crystal at ESRF beamline ID23-2 at 100 K [26]. Data were integrated and scaled with XDS [27]. The crystals belong to the space group *P*6_2_ or *P*6_4_. Initial attempts to solve the structure by molecular replacement, as implemented in Phaser [28] and MOLREP [29], using *Aspergillus nidulans* (*an*) or *Saccharomyces cerevisiae* (*sc*) Rpf2-Rrs1 complex as a search model failed (PDB-IDs: 5BY8, 4XD9, 5A53 [30–32]). Subsequently we sequence adapted the search models using standard procedures as implemented in CHAINSAW, SCULPTOR [33] and MODELLER [34] and combined the resulting models with manual trimming. In addition we took into account the pseudo-symmetrical nature of the brix-fold and tried searching with two separate ‘domains’. Despite extensive trials no clear solution was found. We concluded that the low sequence identity between *ct*Imp4 and *an*Rpf2 and *sc*Rpf2 (both ~17%) might suggest larger structural differences between search and target model causing molecular replacement to fail. Ultimately the structure was solved by molecular replacement using the mr_rosetta [35] pipeline in PHENIX [36]. Placement of *an*Rpf2-*an*Rrs1 [30] in the space group *P*6_4_ resulted in a log-likelihood gain (LLG) of 29.90, indicating a non-solution and similar to the results obtained with our initial molecular replacement trials. However the subsequent rebuilding and relaxation with the ROSETTA pipeline led to a better model, which improved to the LLG to 266.25, indicating a clear solution. After multiple rounds of automated building the Rfree dropped to 27%. The map clearly showed new features and allowed further manual model building. Refinement was carried out with REFMAC5 [37] from the CCP4 package [38] and PHENIX. The final structure contains one *ct*Imp4-*ct*Mpp10 complex in the asymmetric unit. Data collection and refinement statistics are summarized in S3 Table. Comparison of several initial MR solutions with the final model indicated that although the molecule had been correctly placed, the large RMSD (2.35 Å for 182 residues and 2.56 Å for 197 residues, *an*Rpf2-*an*Rrs1 and *sc*Rpf2-*sc*Rrs1 respectively) between search and target model combined with very low sequence identity resulted in very poor starting phases, preventing manual or automatic model building.

Interface residues were identified with the CONTACT program in the CCP4 suite (S4 Table) [38]. Per-residue conservation score was calculated with the ConSurf server [39].

### Sucrose gradient ultracentrifugation

Flag-peptide eluates (max. 500μl) of tandem affinity purifications were further resolved by sucrose gradient ultracentrifugation. Therefore, 95% of the eluted yeast samples were loaded onto a linear 15%–40% (w/v) sucrose gradient containing 50 mM Tris-HCl (pH 7.5), 150 mM NaCl, 1.5 mM MgCl2, 0.001% v/v NP-40, and 1 mM DTT and centrifuged for 16 hours at 129,300 xg and 4°C. Sucrose gradients were made using the Gradient Master device (BioComp Instruments). Fractions from the sucrose gradient were harvested by using the Foxy Junior^®^ fraction collector and pooled to final fractions of ~1.2 ml. The fractions were TCA precipitated and analyzed SDS-PAGE and Coomassie staining.

### Size exclusion chromatography (SEC)

Protein eluates were analyzed by size exclusion chromatography (SEC). Elution fractions were concentrated using a centricon (Amicon Ultra-4, cellulose) to the desired volume before loading on a SEC column if required. Proteins were separated on a Superdex 200 10/300 GL column attached to the Äkta Purifier System (GE Healthcare) in a buffer containing 20 mM HEPES pH 7.5, 150 mM NaCl, 1% v/v glycerol, and 0.001% v/v NP-40 according to manufacturer’s recommendations. As gel filtration standard, a mix of thyroglobulin, bovine γ-globulin, chicken ovalbumin, equine myoglobin, and vitamin B12 was used (Biorad). Fractions of ~500μl were collected, TCA precipitated, and analyzed by SDS-PAGE/Coomassie staining.

## Results

### Utp3/Sas10 and Rps5/uS7 associate with the Mpp10 module

**Fig 1:**
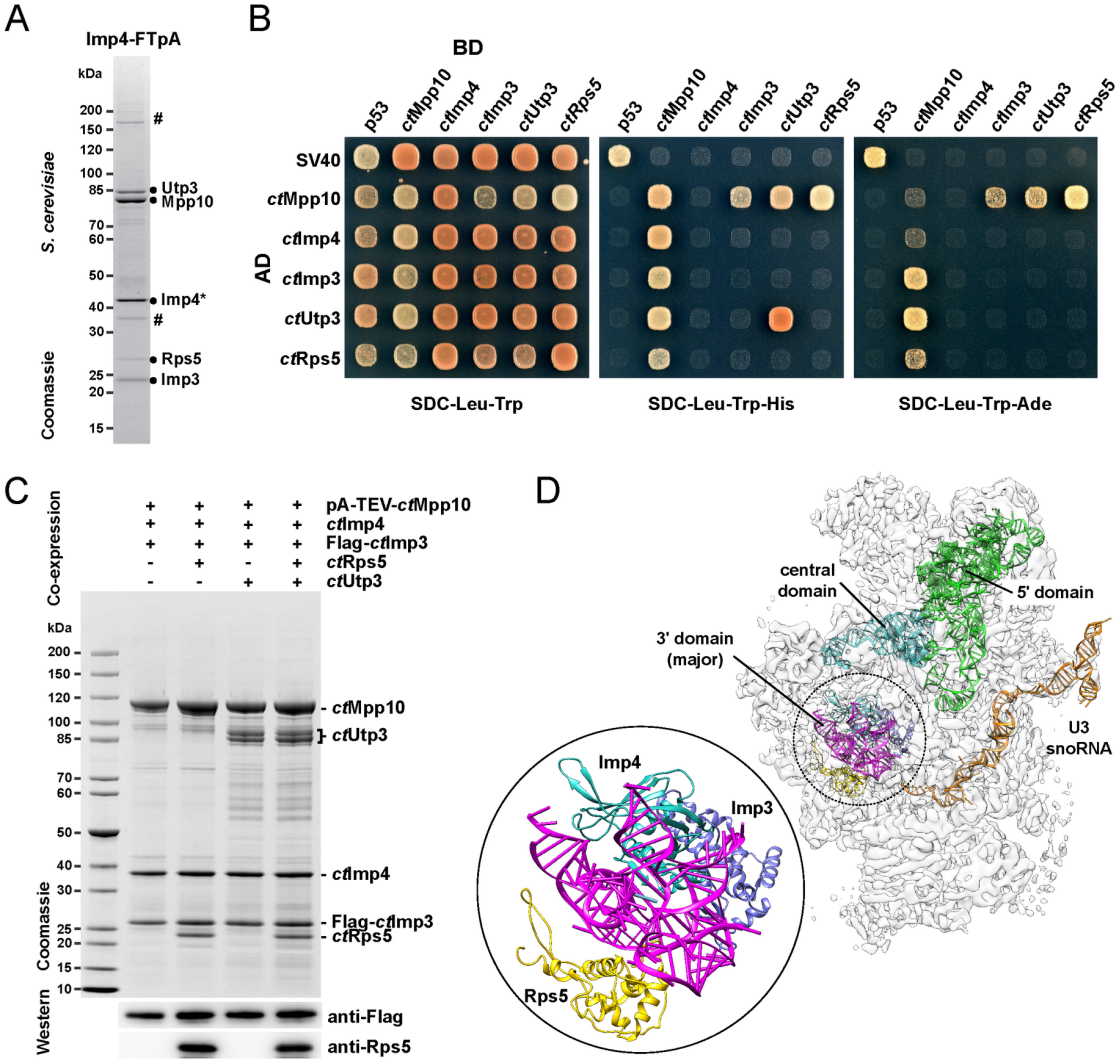
Assembly factor network and reconstitution of the *ct*Mpp10 module. (A) One of the top fractions derived from 15–40% sucrose gradient ultracentrifugation containing the free pool of the yeast Mpp10 complex and associated factors, analyzed by SDS-PAGE and Coomassie staining. The sample was first tandem affinity-purified via yeast Imp4-FTpA (Mpp10 factor) and subsequently resolved by sucrose gradient ultracentrifugation. The labeled proteins were identified by mass spectrometry. Degradation products of Imp4 and dimeric version of Mpp10 are marked with a hashtag (#). (B) Systematic Yeast-2-hybrid analysis of core and associated *ct*Mpp10 assembly factors. The indicated constructs were N-terminally fused to either GAL4 activation domain (AD) or GAL4 binding domain (BD). Yeast transformants were spotted onto SDC-Leu-Trp (permissive), SDC-Leu- Trp-His (selective), and SDC-Leu-Trp-Ade plates (selective for only strong interactions) and grown for 3 days at 30°C. (C) Recombinant co-expression and split tandem-affinity-purification (via pA-TEV-*ct*Mpp10 and Flag-*ct*Imp3) of the *ct*Mpp10 core complex with stepwise addition of associated factors Utp3 and Rps5/uS7. The complexes were assembled by over-expression of indicated protein combinations in yeast (pGAL, high-copy plasmids). Shown are the corresponding Flag-peptide eluates analyzed by SDS-PAGE and Coomassie staining and Western blotting labeled bands were identified by mass spectrometry. Tags were fused to the N-terminus of the corresponding proteins. (D) Position of the *ct*Mpp10 factors *ct*Imp3 and *ct*Imp4 within the 90S cryo-EM density revealing their relative position to the U3 snoRNA (orange) and pre-18S rRNA domains (green, cyan, and purple). Density map of the *C*. *thermophilum* 90S pre-ribosome (EMDB: EMD-8143) and respective fitted *ct*Mpp10 complex members (PDB: 5JPQ) are visualized using Chimera. A magnification of the fitted models for *ct*Imp3 (blue), *ct*Imp4 (turquois), and *ct*Rps5/uS7 (yellow) is shown in a circle, illustrating their close neighborhood within the particle.

As a first step to further explore the role of the Mpp10 module in the context of ribosome biogenesis, we searched for additional factors that associate with the trimeric Mpp10/Imp3/Imp4 complex. Toward this end, the yeast chromosomal IMP4 locus was modified to encode a C-terminally **F**lag-**T**EV-**p**rotein**A**-tagged Imp4 protein. Imp4-FTpA was purified from yeast via tandem-affinity purification followed by sucrose gradient ultracentrifugation. One of the top fractions obtained from the gradient yielded a pentameric complex that, in addition to Mpp10 and its *bona fide* binding partners Imp3 and Imp4, contained also the biogenesis factor Utp3 and the ribosomal protein Rps5 (Fig 1A). Mpp10 and Utp3, with a predicted molecular mass of 67 and 70 kDa, respectively, migrate abnormally slowly in a SDS-polyacrylamide gel, which is typical for acidic proteins [40].

U3 snoRNA-associated protein Sas10, also known as Utp3, was first identified in a screen for yeast genes that, when overexpressed, suppressed silencing of transcriptionally repressed chromatin [41]. It contains the bioinformatically defined SAS10/C1D domain, an 80 amino acid residues α-helical domain, that is present in two other yeast proteins, namely Lcp5 (another 90S factor) and Rrp47 (exosome-binding factor) [42]. The identification of Utp3 as Mpp10-associated factor is in agreement with previously published data reporting positive Yeast-2-Hybrid (Y2H) interaction between these two proteins [43, 44]. Rps5 is an essential protein located at the back of the 18S rRNA ‘head’ domain in the mature small subunit [45]. In fact, Rps5 is required for the correct assembly of other SSU head domain ribosomal proteins and therefore for the overall folding of the 3’ major domain of the mature 18S rRNA [46]. Moreover, depletion of this protein completely abolishes the nuclear export of the nascent 40S particles and causes a delay in early 18S rRNA processing events with a subsequent accumulation of early pre-rRNA species [47].

To dissect the binding properties of Utp3 and Rps5 to the Mpp10-Imp3-Imp4 complex, a systematic Y2H analysis was performed using the respective orthologs from *C*. *thermophilum (ct)* as thermophilic proteins have previously been shown to have better biochemical and biophysical characteristics in structural biology than their mesophilic counterparts [7, 48]. *Ct*Imp3, *ct*Imp4, *ct*Utp3, and *ct*Rps5 all interact with *ct*Mpp10, whereas among these factors no interaction was detected, suggesting that Mpp10 provides a platform for its multiple binding partners (Fig 1B).

Next, we sought to reconstitute the Mpp10 module, using the *C*. *thermophilum* orthologs, by heterologous co-expression of the different members of the complex in *S*. *cerevisiae*, each under control of the inducible *GAL* promoter, followed by split-tag tandem affinity purification, using pA-TEV-*ct*Mpp10 and FLAG-*ct*Imp3 as first and second bait, respectively. We started by successfully reconstituting the core complex carrying pA-TEV-*ct*Mpp10, FLAG-*ct*Imp3 and untagged *ct*Imp4. Subsequently, in addition to the core complex members, we co-expressed also *ct*Utp3, *ct*Rps5 or both. In all cases, we were able to reconstitute the corresponding tetrameric and pentameric complexes. Notably, binding of *ct*Rps5 and *ct*Utp3 to the Mpp10 core complex was independent from each other (Fig 1C). Mpp10 is predicted to be a rather flexible protein with large coiled-coil regions, whose exact location in the 90S has so far not been determined. Interestingly, the early-binding ribosomal protein *ct*Rps5 is localized in the vicinity of both *ct*Imp3 and *ct*Imp4 in the *ct*90S particle, suggesting that Mpp10 might be connecting these factors in that area (Fig 1D) [7].

Taken these findings together, we could identify two additional binding partners of the Mpp10 module, the biogenesis factor Utp3 and the ribosomal protein Rps5. Furthermore, our data indicate that Mpp10 provides a shared binding platform establishing simultaneous interactions with all its binding partners.

### Utp3 interacts directly with the Mpp10 N-terminus

Consistent with our previous findings, the coiled-coil regions of Mpp10 have been suggested to be involved in multiple intra and/or intermolecular interactions [49]. Therefore, we set out to identify an Mpp10 minimal binding motif required for interaction with Utp3. In yeast and human the binding regions for Imp3 and Imp4 have already been reported [49, 50]. Based on these data and multiple sequence alignments and secondary structure prediction, *ct*Mpp10 was divided into five consecutive domains: 1) an N-terminal domain (residues 1–162), 2) a long disordered acidic loop region (residues 184–467), 3) Imp4 interaction domain (residues 475–562), 4) Imp3 interaction domain (residues 612–643) and 5) a C-terminal region rich in basic residues (673–785) (S1 Fig).

**Fig 2:**
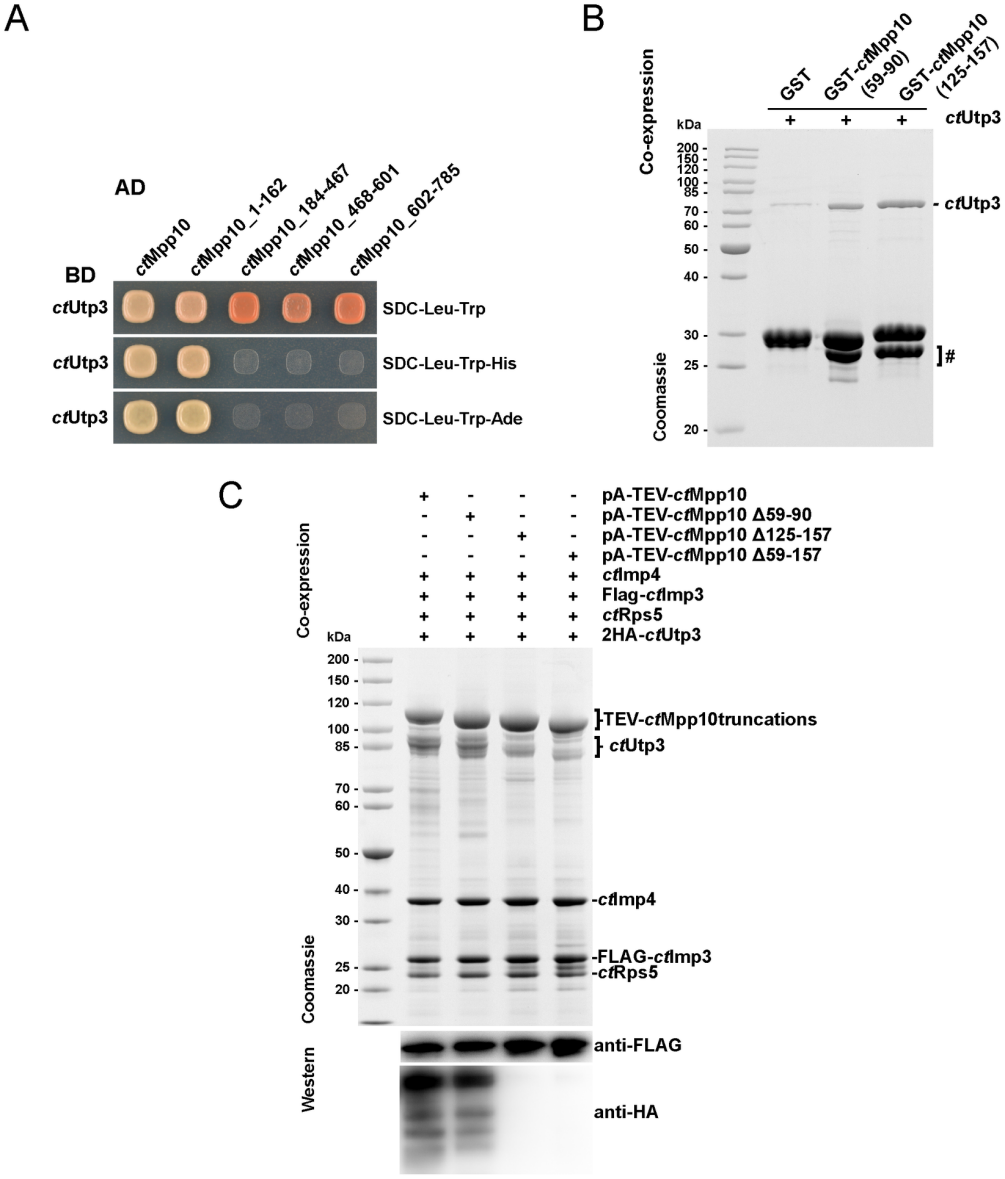
Two hydrophobic patches within the N-terminal region mediate the direct interaction between Mpp10 and Utp3. (A) Yeast 2-hybrid analysis of full-length *ct*Utp3 tested with four indicated fragments of *ct*Mpp10. The indicated constructs were N-terminally fused to either GAL4 activation domain (AD) or GAL4 binding domain (BD). Yeast transformants were spotted onto SDC-Leu-Trp, SDC-Leu-Trp-His, and SDC-Leu-Trp-Ade plates and grown for 3 days at 30°C. (B) Recombinant GST and GST-*ct*Mpp10 truncations were co-expressed with *ct*Utp3 in *E*. *coli*, and subsequently bound to glutathione resin. GSH-eluates were analyzed by SDS-PAGE followed by Coomassie staining. Labeled bands were identified by mass spectrometry. Lower bands correspond to degradation products and are marked with a hashtag (#). (C) Recombinant overexpression of the *C*. *thermophilum* Mpp10 complex components in *S*. *cerevisiae*, followed by split-tag affinity-purification using pA-TEV-*ct*Mpp10 and Flag-*ct*Imp3, as first and second bait, respectively. The complexes were assembled by over-expression of indicated protein combinations in yeast (pGAL, high-copy plasmids). In this case, we additionally used an N-terminally HA tagged version of *ct*Utp3. FLAG eluates were subject to SDS-PAGE followed by Coomassie staining and Western blot analysis using anti-HA and anti-FLAG antibodies.

To pinpoint the region of Mpp10 mediating interaction with *ct*Utp3, we carried out an initial Y2H assay using full-length *ct*Utp3 and tested binding with a series of *ct*Mpp10 truncations. Neither the acidic loop region (residues 184–467) nor the Imp4 binding domain (residues 468–601) showed detectable binding to *ct*Utp3. Moreover, a fragment containing both Imp3 binding region and the C-terminus of *ct*Mpp10 (residues 602–785) also failed to bind *ct*Utp3. *ct*Utp3 binds exclusively within the N-terminus of *ct*Mpp10 corresponding to residues 1–162 (Fig 2A). To further narrow down *ct*Mpp10 to a minimal motif, we tested two truncations that include two conserved hydrophobic patches present in *ct*Mpp10 N-terminal region: *ct*Mpp10 (59–90) and *ct*Mpp10 (125–157) (S1 Fig). Recombinant GST, GST-*ct*Mpp10 (59–90) and GST-*ct*Mpp10 (125–157) were co-expressed with His-*ct*Utp3 in *E*. *coli*, immobilized on glutathione beads and then eluted with glutathione. Both GST-*ct*Mpp10 (59–90) and GST-*ct*Mpp10 (125–157), but not GST alone, were able to interact with His-*ct*Utp3, indicating that each motif is sufficient for Utp3 binding (Fig 2B).

To obtain further insight into the importance of these discrete motifs in binding to Utp3, we again took advantage of our reconstitution system. We then co-expressed Mpp10 constructs lacking one or both binding motifs. Interestingly, pA-TEV-*ct*Mpp10 (Δ59–90) is still able to interact with 2HA-*ct*Utp3. In contrast, interaction is completely abolished upon deletion of the second minimal motif *ct*Mpp10 (Δ125–157) and *ct*Mpp10 (Δ59–157), suggesting that the motif corresponding to residues 125–157 motif predominantly mediates this interaction (Fig 2C).

Taken together, our findings indicate that Utp3 binds directly to the N-terminal domain of Mpp10. Two conserved motifs within *ct*Mpp10 (residues 59–90 and 125–157) are able to mediate this interaction but only the second motif is essential for binding.

### Mpp10 interaction with Rps5 requires the acidic loop region

**Fig 3:**
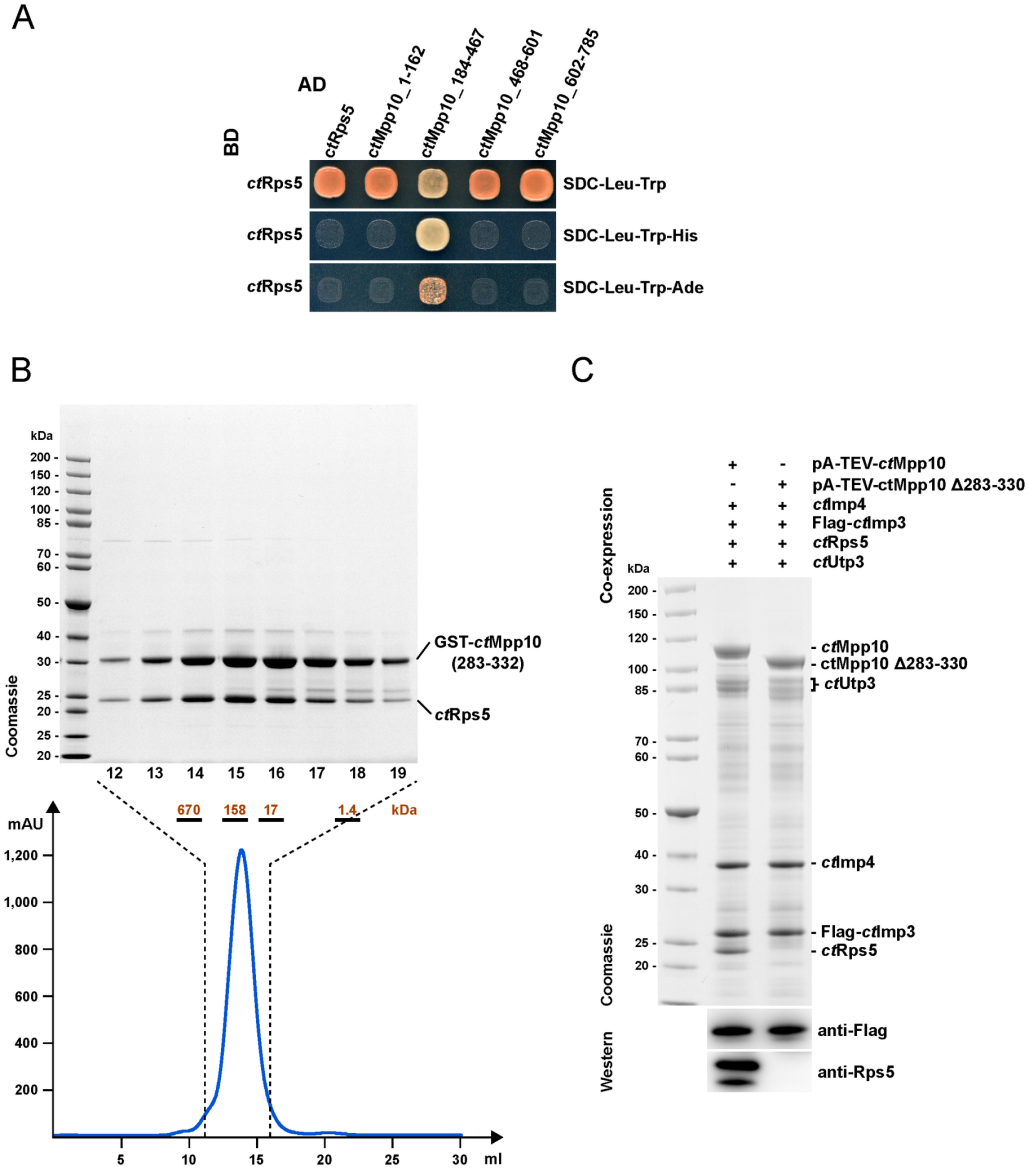
Identification of the minimal Rps5-binding motif within Mpp10. (A) Yeast 2-hybrid analysis of full-length *ct*Rps5 tested with four indicated fragments of *ct*Mpp10. The indicated constructs were N-terminally fused to either GAL4 activation domain (AD) or GAL4 binding domain (BD). Yeast transformants were spotted onto SDC-Leu-Trp, SDC-Leu-Trp-His, and SDC-Leu-Trp-Ade plates and grown for 3 days at 30°C. (B) Size exclusion chromatography (SEC) of the reconstituted GST-*ct*Mpp10(283–332)-*ct*Rps5 heterodimer. Based on the elution profile recorded at 280 nm, we analyzed fractions 12–19 by SDS-PAGE and Coomassie staining. The complete elution profile (in ml) using a Superdex 200 10/300 column is shown underneath, with indication of the region corresponding to fractions 12–19. The complex was recombinantly co-expressed in *E*. *coli*, purified via GST pull-down and eluted with glutathione (GSH). Labeled bands were verified by mass spectrometry. (C) Assembly of the Mpp10 module by recombinant overexpression of the *C*. *thermophilum* counterparts, in *S*. *cerevisiae*, followed by split-tag affinity-purification using pA-TEV-*ct*Mpp10 and Flag-*ct*Imp3, as first and second bait respectively. The complexes were assembled by over-expression of indicated protein combinations in yeast (pGAL, high-copy plasmids). FLAG eluates were subject to SDS-PAGE followed by Coomassie staining and Western blot analysis using anti-Rps5 and anti-FLAG antibodies.

Using an Y2H approach, similar to the one used for Utp3, we identified the acidic loop, *ct*Mpp10 (184–467), as the region required for Mpp10 interaction with Rps5 (Fig 3A). We defined 5 different regions within the acidic loop that were tested to determine the minimal motif required for Rps5 binding by Mpp10 (S2 Fig). Y2H assays allowed the exclusion of regions 1, 4 and 5 as the binding motifs (data not shown). Two different fragments, one containing regions 2 and 3 (GST-*ct*Mpp10 (283–332)) and other containing regions 2, 3 and 4 (GST-*ct*Mpp10 (283–410)) were co-expressed with His-*ct*Rps5. In both cases, we observed the formation of a complex between Mpp10 truncation and His-*ct*Rps5 (S2 Fig). The obtained stoichiometric complex between GST-*ct*Mpp10 (283–332) and His-*ct*Rps5 (calculated mass of ~58 kDa) remained stable during size exclusion chromatography corroborating this solid interaction (Fig 3B). A protein size standard run on the same column suggests that this complex runs a dimer during gel filtration, most likely due to GST dimerization.

Subsequently we attempted to confirm that deletion of this region would be sufficient to abolish binding between *ct*Rps5 and *ct*Mpp10. While full-length *ct*Mpp10 co-purified all members of the Mpp10 complex, *ct*Mpp10 Δ283–330 could no longer co-purify *ct*Rps5 (Fig 3C). For this mutant, we also observe a decrease in the levels of *ct*Utp3 co-purifying with the Mpp10 complex. One possible explanation is that by truncating the protein, even though not truncating the region responsible for Utp3 binding, we might be creating space constraints that affect the interaction of Mpp10 N-terminus with Utp3.

Based on these findings, we conclude that ctRps5 binds to Mpp10 within its acidic loop region and restricted the interaction site to an acidic stretch in Mpp10 corresponding to residues 283–330. Deletion of these residues specifically hampers interaction between *ct*Mpp10 and *ct*Rps5.

### Structure of the *ct*Imp4-*ct*Mpp10 complex

**Fig 4:**
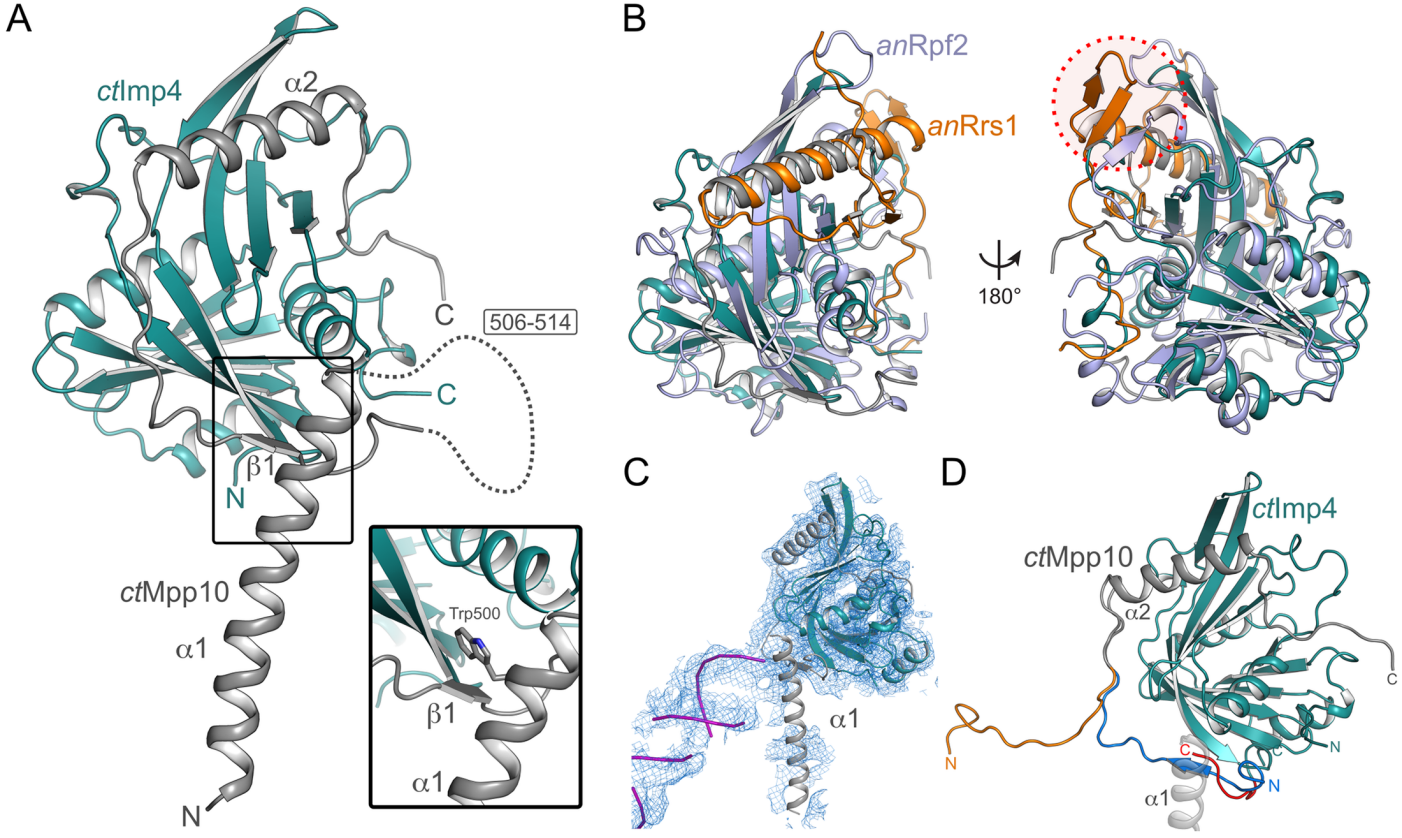
Crystal structure of *ct*Imp4-*ct*Mpp10 complex. (A) Overall structure of the *ct*Imp4-*ct*Mpp10 complex. The BRIX domain of *ct*Imp4 (teal) is completed by helix α2 of *ct*Mpp10 (grey). Helix α1 of *ct*Mpp10 interacts via Trp500 with *ct*Imp4 (inset). For the sake of clarity *ct*Mpp10 helix α1 is omitted in the other panels. (B) Comparison of *ct*Imp4-*ct*Mpp10 with *an*Rpf2-*an*Rrs1 (PDB-ID: 5BY8 [30]) The overall fold between the BRIX domain proteins *ct*Imp4 (teal) and *an*Rpf2 (light-blue) and their ligands *ct*Mpp10 (grey) and *an*Rrs1 (orange) is preserved. Beta-augmentation on the C-terminal sub-domain of the BRIX protein is only observed in the *an*Rpf2-*an*Rrs1 complex (red circle). (C) Rigid body docking of the *ct*Imp4-*ct*Mpp10 complex into the 5.1 Å map of the yeast 90S particle [8]. While the BRIX domain of *ct*Imp4 (teal) and *ct*Mpp10 helix α2 (grey) are completely covered by electron density, helix α1 of *ct*Mpp10 is not. This suggests that in context of the 90S, this helix occupies another location. The tip of helix α1 is occupied by a RNA double helix (purple) in the yeast 90S density. (D) Alternative modeling of the termini of *ct*Imp4 and *ct*Mpp10 based on the crystal structure and cryo-EM density. In the crystal structure (grey/blue), the N-terminus (residues 515–530) of our *ct*Mpp10 construct interacts with *ct*Imp4, whereas when modeled based on the cryo-EM density it extends away (orange) from *ct*Imp4. Likewise the C-terminus of *ct*Imp4 can be extended and modeled (red) into the now remaining cavity between *ct*Mpp10 helix α1 (grey ribbon) and *ct*Imp4.

To obtain atomic insight into the interaction network of Mpp10, we tried to crystallize complexes between Mpp10 and its cognate binding partners. We reconstituted a minimal complex of *ct*Imp4 (residues 75–274) and *ct*Mpp10 (residues 433–562) and determined its crystal structure to a resolution of 1.88 Å (Fig 4A). Residues 433–474 as well as 506–514 of *ct*Mpp10 are not resolved in the crystal structure.

The overall fold of the *ct*Imp4-*ct*Mpp10 complex is very similar to that of Rpf2-Rrs1 from *Aspergillus nidulans* (*an*) and *Saccharomyces cerevisiae* (*sc*), despite the rather low sequence identity of ~17%. *Ct*Imp4 exhibits the typical BRIX fold [30–32, 51], comprised of two anti-codon binding domains, of which one is completed by the brix-ligand, by an α-helix provided by the brix-ligand, *ct*Mpp10 in this case (S1 Fig). This helix contains several conserved charged (Glu536, Glue542 and Arg548) as well as hydrophobic residues (Ile545 and Ile549) (S1 Fig). There are several local structural differences when compared to Rpf2-Rrs1, leading to a RMSD of 2.35 Å for 182 residues (*A*. *nidulans*, Fig 4B*)*, 2.56 Å for 197 residues (*S*. *cerevisiae*, S3 Fig). Furthermore, there is no beta-augmentation on the C-terminal subdomain of *ct*Imp4 as observed in Rpf2-Rrs1, limiting the domain complementation to helix α2 (S3B Fig). In addition, *ct*Mpp10 also makes several contacts with the N-terminal subdomain of *ct*Imp4. Interaction at the N-terminal subdomain includes the short beta-strand β1, which is sandwiched between *ct*Imp4 and helix α1 of *ct*Mpp10. A striking difference to the Rpf2-Rrs1 complex is the presence of a long α-helix (α1) at the N-terminus of our *ct*Mpp10 construct, which interacts via a tryptophan residue (Trp500) with *ct*Imp4 at the interface between the two subdomains of *ct*Imp4 (Fig 4A, inset). In order to understand if the position of *ct*Mpp10 helix α1 represents a physiologically relevant conformation or is stabilized by crystal packing, we performed rigid body docking of our crystal structure into the previously published cryo-EM density of the 90S particle from *C*. *thermophilum* [7] as well as *S*. *cerevisiae* [8, 9]. While the completed Brix domain results in an excellent fit to the density, there is no corresponding density for helix α1 of *ct*Mpp10 in any of the 90S pre-ribosomal particles [7–9]. In fact, the density at this position in the 90S belongs to a RNA double helix and has been partially modeled previously ([9] and Fig 4C). Furthermore, we could trace 7 residues of the *ct*Imp4 C-terminus (which had been omitted for crystallization) within the 5.1 Å cryo-EM map of the yeast 90S particle. A tryptophan (Trp282) residue at the very C-terminus of *ct*Imp4 would locate to the same position as the tryptophan of helix α1 of *ct*Mpp10 (Fig 4D), suggesting that the current conformation may be the result of truncation and crystal packing. Further comparison of the cryo-EM map and our crystal structure suggests that *ct*Mpp10 residues 515–530, locate towards other proteins within the 90S particle and do not participate in beta-augmentation with *ct*Imp4 (Fig 4D and S3C Fig).

### *In vivo* analysis of binding-deficient *mpp10* mutants

**Fig 5:**
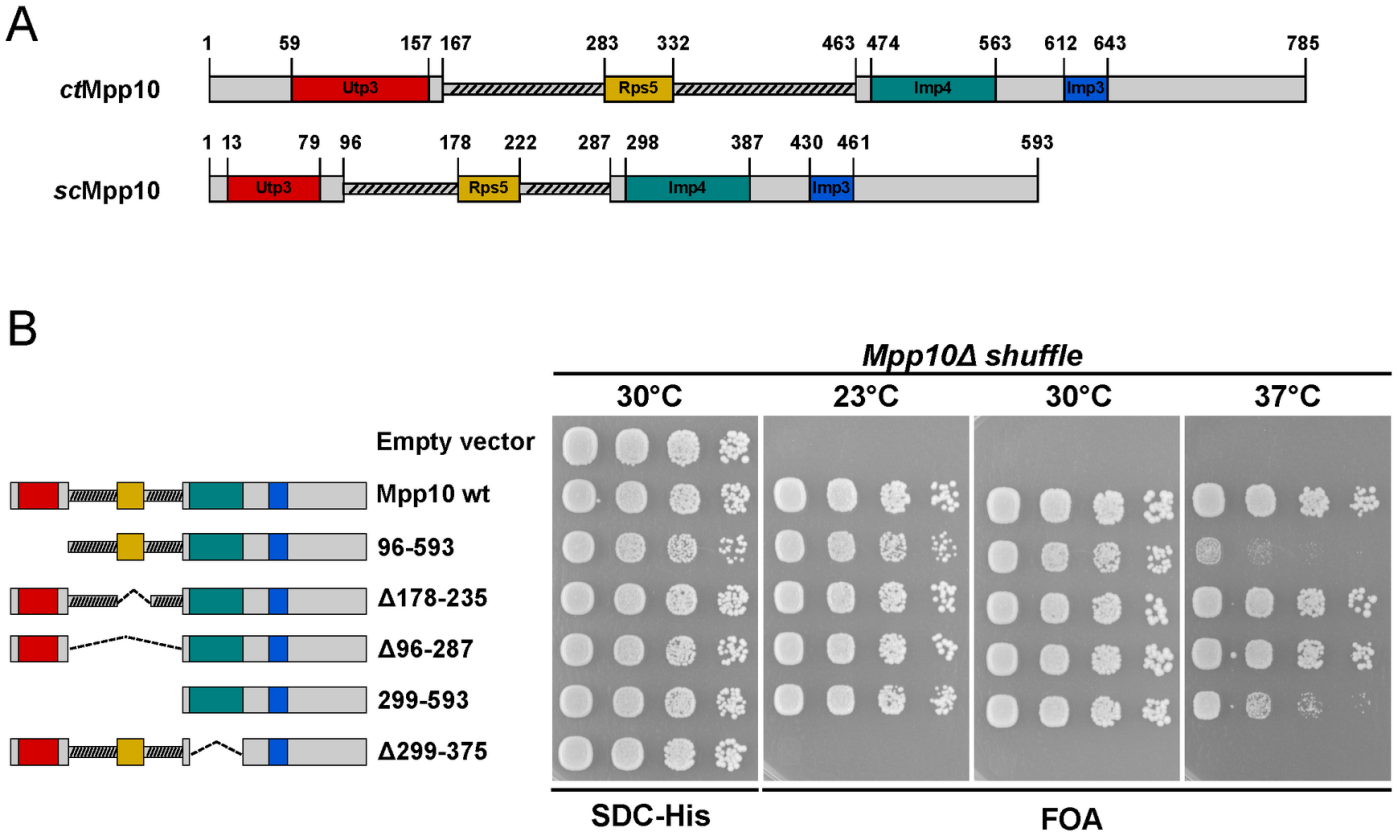
Growth analysis of strains with different Mpp10 domain truncations. (A) Overall domain organization for both *ct*Mpp10 and *sc*Mpp10 including the identified minimal binding motifs for *ct*Utp3, *ct*Rps5, *ct*Imp4, and the published *ct*Imp3 [50]. (B) Growth analysis of yeast *mpp10*Δ shuffle strain complemented with the indicated Mpp10 constructs under *TEF1* promoter control. The *mpp10*Δ shuffle strain was transformed with *HIS3* plasmids carrying wild-type *MPP10* and truncations. Subsequently, the *URA3*-*MPP10* shuffle plasmid was shuffled out on SDC+5-FOA plates. Transformants were spotted in 6-fold serial dilutions onto SDC-His or SDC+FOA. Plates were incubated at the indicated temperatures for 3 days.

To assess the impact of these interactions *in vivo*, we turned to the *Saccharomyces cerevisiae* system and used an *mpp10*Δ shuffle strain for complementation of the otherwise non-viable *mpp10*Δ (null) mutant. Based on our previous data, we located the equivalent regions in *S*. *cerevisiae* necessary for binding Utp3, Rps5 and Imp4 and deleted them (Fig 5A). A mutant containing a truncation of *sc*Mpp10 N-terminal region (*sc*Mpp10 96–593), the region responsible for binding Utp3, displays a slow growth phenotype, especially at 37°C (Fig 5B). Interestingly, a previously reported *mpp10-3* mutant, consisting of a 46 amino acids N-terminal truncation, displays no growth phenotype at any of the tested temperatures [52]. This truncation is lacking our reported Utp3 minimal binding site 1 that we have found to be sufficient, but not required for binding Utp3 (Fig 2C and S1 Fig), providing an explanation for the lack of a growth defect. Interestingly, the *mpp10* mutant lacking the Rps5 binding region, was viable displaying a normal growth (*sc*Mpp10 (Δ178–235)), suggesting that the interaction between Rps5 and Mpp10 is not essential. This was also observed for a mutant with the complete deletion of the acidic loop, *sc*Mpp10 (Δ96–287). Intriguingly, deletion of both N-terminal and acidic loop regions (*sc*Mpp10 (299–593)) partially rescued the *ts* phenotype of the *sc*Mpp10 (96–593) mutant at 37°C. Furthermore, deletion of the Imp4 binding region (*sc*Mpp10 (Δ299–375)) exhibited lethality in all of the tested temperatures (Fig 5B). Indeed, Mpp10 has been reported to be essential for the stability of both Imp3 and Imp4 presumably by binding them [53].

## Discussion

The heterotrimeric Mpp10-Imp3-Imp4 complex is an essential 90S module, which is localized at the core of the 90S pre-ribosome adjacent to the UTP-B complex. Imp3 and Imp4, which have been implicated in controlling the formation of pre-rRNA::U3 RNA hybrids during 90S maturation [18], were localized within the *ct*90S cryo-EM map based on their homology to ribosomal protein uS4 and the Brix-domain, respectively. Recent reports have found small helical segments associated with both Imp3 and Imp4 that have been suggested to be Mpp10 [7–9]. Our crystal structure reveals a long α-helix (α1, residues 475–505) of *ct*Mpp10, which is anchored via Trp500 to *ct*Imp4 and is not part of the canonical helix (α2) that docks with the BRIX fold. Unambiguous assignment of this helix to the helical densities observed in cryo-EM 90S densities will however require higher resolution. In addition to the known interaction with Imp3 and Imp4, in this study, we have identified Utp3/Sas10 and ribosomal protein Rps5/uS7 as additional binding partners of Mpp10 and mapped their respective binding regions within Mpp10. In the 90S particle, ribosomal protein S5 has been found to bind in the vicinity of both Imp3 and Imp4. However, Utp3, presumably a rather flexible protein, could not be located in any of the 90S structures published to date, although SDS/PAGE and mass spectrometry have shown that it is a component of the particle [7–9]. Based on our data we hypothesize that Utp3 contacts Mpp10’s N-terminal region. We propose that Mpp10, positioned at the 90S core, provides an assembly scaffold with linear interaction motifs for the different members of this pentameric module.

Ribosome biogenesis is a tightly regulated process, during which ribosomal proteins are synthesized in the cytoplasm and then imported into the nucleolus where they associate with pre-ribosomal particles. However, many ribosomal proteins have been shown or suggested to be highly unstable and prone to aggregation. Furthermore, they constitute one of the most common ubiquitinated substrates in the cell, being readily subject to degradation by the ubiquitin-proteasome system when in an unbound state in the cell [54–56]. Several different reports have unveiled the existence of chaperones responsible for ensuring the correct and timely incorporation of the ribosomal [57–61]. Similarly, Mpp10 may represent one of these dedicated chaperones, ensuring the correct incorporation of Rps5 into the particle. However, deletion of the Rps5 binding motif of Mpp10 does not display a growth defect suggesting a non-essential role for this interaction. Indeed, several of these dedicated chaperones are non-essential and there are alternative and redundant pathways and factors involved in the incorporation of the ribosomal proteins into the nascent 90S pre-ribosome [57–62]. Interestingly, the cryo-EM structure of the *ct*90S particle suggests that Rps5 has additional physical contacts to the Emg1 homodimer and a so far UTP-A unassigned beta-propeller that could also facilitate Rps5 recruitment [7]. Alternatively, association of Rps5 with its canonical rRNA binding site within the pre-40S head domain might already be sufficient for its incorporation. Notably, Mpp10 possesses a putative nuclear localization sequence (NLS) within its C-terminal region, and it has been shown that formation of a ternary complex between Mpp10, Imp3 and Imp4 is essential for their nucleolar localization [49, 63]. One interesting possibility is that Mpp10 could additionally mediate the active import of Rps5 into the nucleus. In agreement with this idea, we used a non-radioactive pulse-chase analyses combined with affinity-purification, and found that newly synthesized *sc*Mpp10 co-precipitated with Srp1/Kap60, a karyopherin that mediates the nuclear import of cargos, together with Kap95 [64]. This interaction was lost upon deletion of Mpp10’s C-terminal region, where the putative NLS is localized (data not shown).

Interestingly deletion of Mpp10’s Utp3 binding motif is deleterious to the cell, suggesting a putative functional significance for this interaction. Mpp10 complex association with the 90S particle depends on previous incorporation of the UTP-B complex [5, 6, 65]. Furthermore, Utp3 has been suggested to be the link between the Mpp10 module and the UTP-B complex. Indeed, Y2H analysis revealed interactions between Utp3 and the members of UTP-B complex Utp6 and Utp21 [43] and we have confirmed the interaction between Utp3 and Mpp10 even pinpointing two regions in the Mpp10 N-terminal domain able to bind Utp3. Several possibilities arise for the possible function of the observed interaction. Similarly to what we proposed for Rps5, Mpp10 interaction might be important also for nuclear import and/or proper incorporation of Utp3 into the 90S particle. Additionally, this interaction could have a functional role in the context of the 90S particle. One possibility is that Utp3, being a rather flexible protein and the bridging factor between the two 90S modules could potentially ensure its proper structural arrangement towards each other. This could contribute for positioning the Mpp10 complex in the vicinity of the area where it mediates the formation of the U3::pre-18SRNA heteroduplexes. Future EM studies might help to reveal where Utp3 is sitting and might shed some more light into its function.

## Supporting information

S1 Fig Multiple sequence alignment for Mpp10. Indicated is the overall Mpp10 domain organization with an N-terminal domain, indicated by a red line below the alignment, with two conserved hydrophobic motifs required for Utp3 binding (inside a dashed line box). Next, we pinpointed the acidic loop, indicated in yellow that contains the Rps5 binding motif (inside a dashed line box), followed by the Imp4 binding region, indicated in green. Then, the Imp3 binding domain based on what was previously described in [50] in blue, followed by the C-terminal region rich in basic residues, in grey. Helix α1 and α2 of *ct*Mpp10 are drawn above the alignment, strictly conserved residues in α2 are marked with red dots. Aligned proteins were chosen based on homology groups obtained for Mpp10 using Homologene. Alignments were performed with T-coffee and visualized with Jalview. Proteins from different species were used: *Chaetomium thermophilum*, *Saccharomyces cerevisiae*, *Kluyveromyces lactis*, *Eremothecium gossypii*, *Schizosaccharomyces pombe*, *Homo sapiens*, *Pan troglodytes*, *Macaca mulatta*, *Canis lupus familiaris*, *Bos Taurus*, *Mus musculus*, *Rattus norvegicus*, *Xenopus tropicalis and Danio rerio*.
(TIF)

S2 Fig Initial mapping of S5 binding region from Mpp10. (A) Recombinant GST and GST *ct*Mpp10 truncations were co-expressed *ct*Rps5 in *E*.*coli*, and subsequently bound to glutathione resin. GSH-eluates were analyzed by SDS-PAGE followed by Coomassie staining. Labeled bands were identified by mass spectrometry. (B) Scheme of the different regions of the acidic loop that were tested for binding.
(TIF)

S3 Fig Comparison of the *ct*Imp4-*ct*Mpp10 complex with archael Mil protein and interaction with Bms1. (A) Comparison of *ct*Imp4-*ct*Mpp10 with *sc*Rpf2-*sc*Rrs1 (PDB-ID: 5A53 [32]). The BRIX fold is conserved between *ct*Imp4 (teal) and *sc*Rpf2 (light-brown). The BRIX-ligands, *ct*Mpp10 (grey) and *sc*Rrs1 (lime-green) occupy the same position via a single α-helix. No β-augmentation is observed for *ct*Mpp10 (red circle). (B) Structure of *ct*Imp4 can be divided into sub-domains, the N- (teal) and C-terminal half (cyan). (C) Comparison of *ct*Imp4 (teal) and the archeal Imp4-like protein Mil (orange). While *ct*Imp4 is completed by *ct*Mpp10 helix α2, no such ligand is known for Mil. (D) Modeling of the *ct*Mpp10 N-terminus (orange) based on the yeast 90S cryo-EM density suggests an interaction with Bms1 (blue).
(TIF)

S1 Table Plasmids used in this study. (PDF)

S2 Table Yeast strains used in this study. (PDF)

S3 Table Data collection and refinement statistics. (PDF)

S4 Table Interface residues, ConSurf score and distance. (XLSX)
