# Correction: Mpp10 represents a platform for the interaction of multiple factors within the 90S pre-ribosome

**DOI:** 10.1371/journal.pone.0234932

**Published:** 2020-06-12

**Authors:** Bebiana Sá-Moura, Markus Kornprobst, Satyavati Kharde, Yasar Luqman Ahmed, Gunter Stier, Ruth Kunze, Irmgard Sinning, Ed Hurt

The following information is missing from the Funding statement: This work was also supported by the European Research Council (ERC) (ADG 741781 GLOWSOME to E.H.).
