# Supplementary figures and images for: Mpp10 represents a platform for the interaction of multiple factors within the 90S pre-ribosome
Source: PLoS One. 2017 Aug 16;12(8):e0183272. doi: 10.1371/journal.pone.0183272 (PMC5558966; doi:10.1371/journal.pone.0183272)

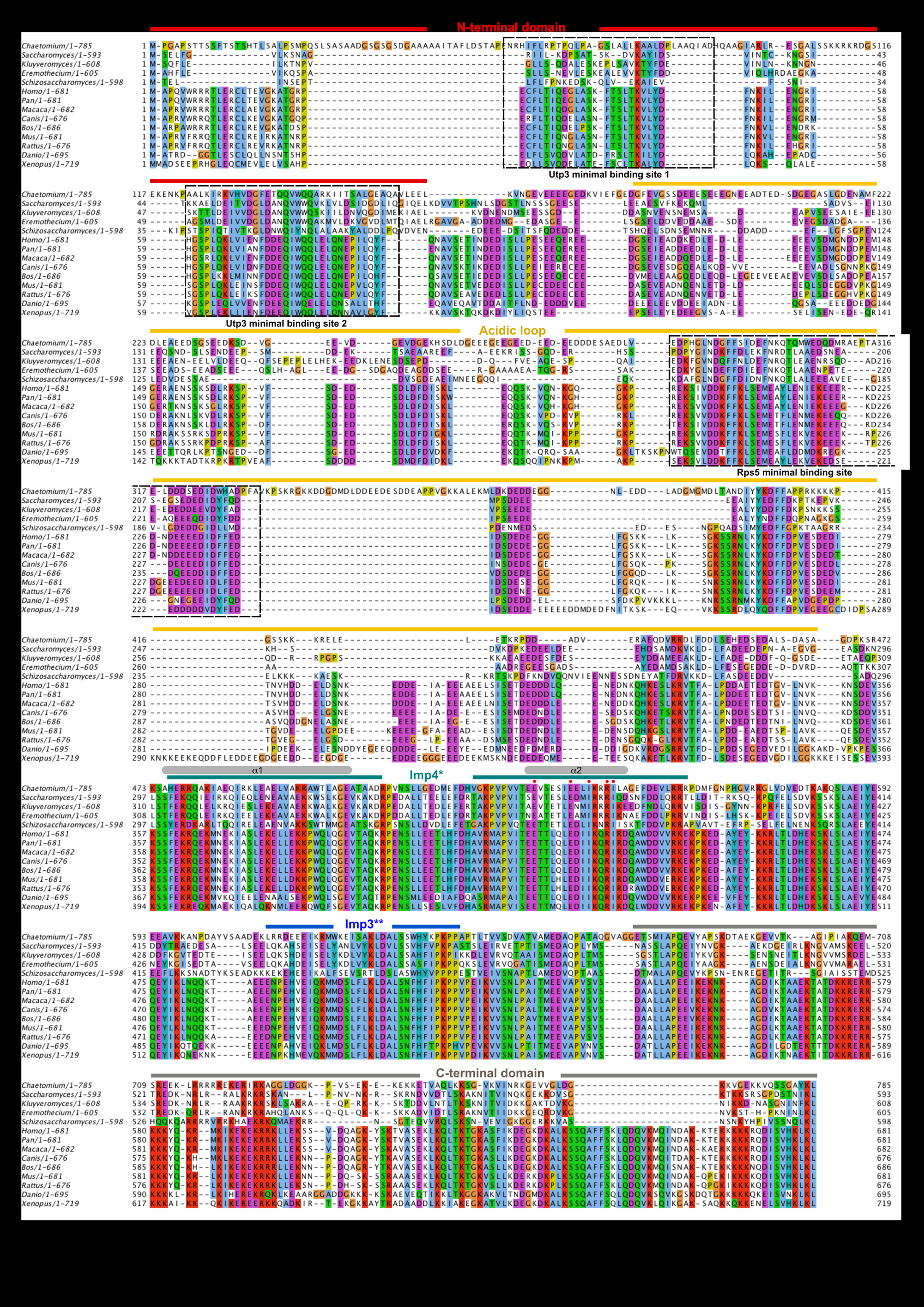

Supplement: S1 Fig — Indicated is the overall Mpp10 domain organization with an N-terminal domain, indicated by a red line below the alignment, with two conserved hydrophobic motifs required for Utp3 binding (inside a dashed line box). Next, we pinpointed the acidic loop, indicated in yellow that contains the Rps5 binding motif (inside a dashed line box), followed by the Imp4 binding region, indicated in green. Then, the Imp3 binding domain based on what was previously described in [50] in blue, followed by the C-terminal region rich in basic residues, in grey. Helix α1 and α2 of ctMpp10 are drawn above the alignment, strictly conserved residues in α2 are marked with red dots. Aligned proteins were chosen based on homology groups obtained for Mpp10 using Homologene. Alignments were performed with T-coffee and visualized with Jalview. Proteins from different species were used: Chaetomium thermophilum, Saccharomyces cerevisiae, Kluyveromyces lactis, Eremothecium gossypii, Schizosaccharomyces pombe, Homo sapiens, Pan troglodytes, Macaca mulatta, Canis lupus familiaris, Bos Taurus, Mus musculus, Rattus norvegicus, Xenopus tropicalis and Danio rerio. (TIF) [file pone.0183272.s001.tif]

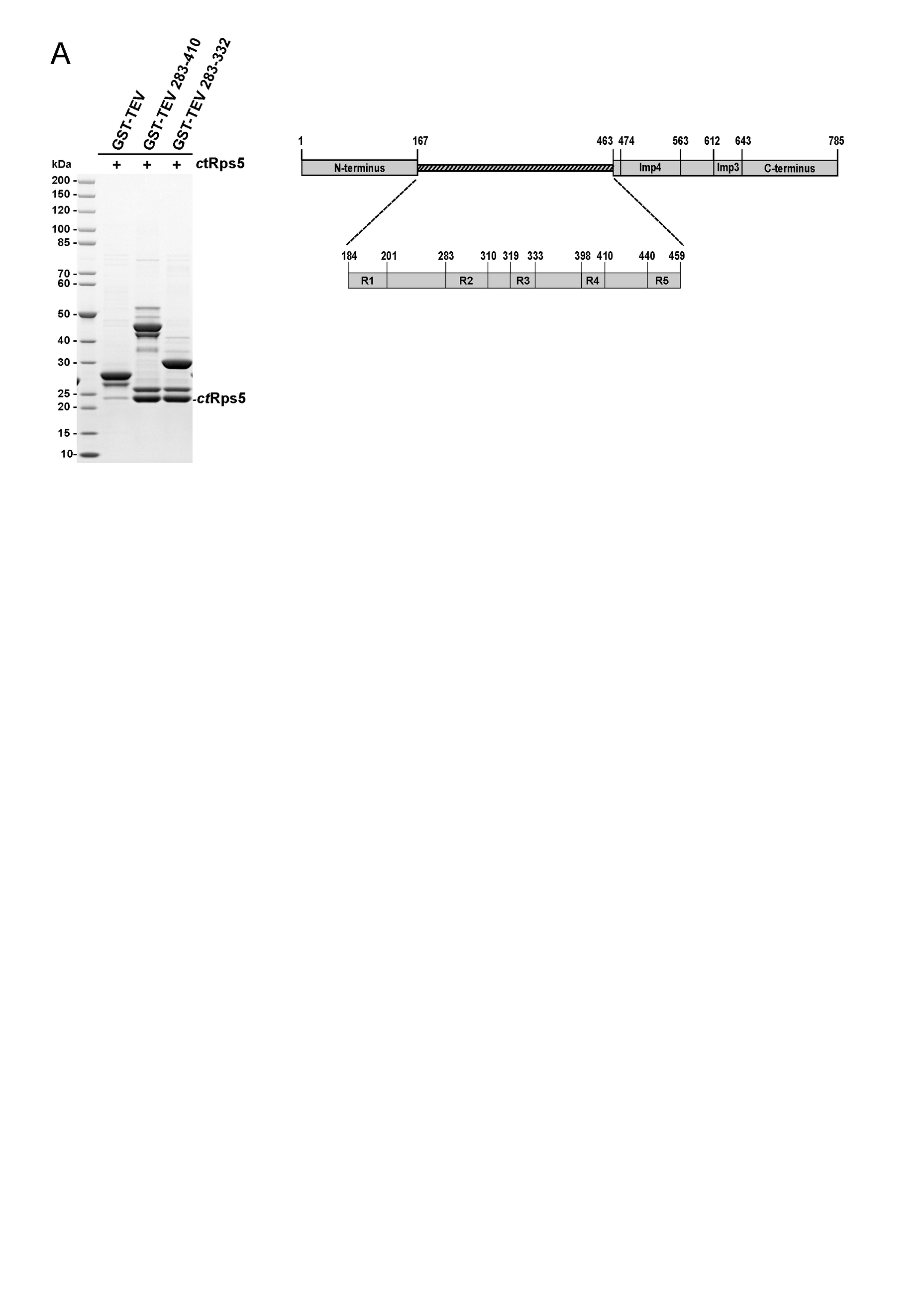

Supplement: S2 Fig — (A) Recombinant GST and GST ctMpp10 truncations were co-expressed ctRps5 in E.coli, and subsequently bound to glutathione resin. GSH-eluates were analyzed by SDS-PAGE followed by Coomassie staining. Labeled bands were identified by mass spectrometry. (B) Scheme of the different regions of the acidic loop that were tested for binding. (TIF) [file pone.0183272.s002.tif]

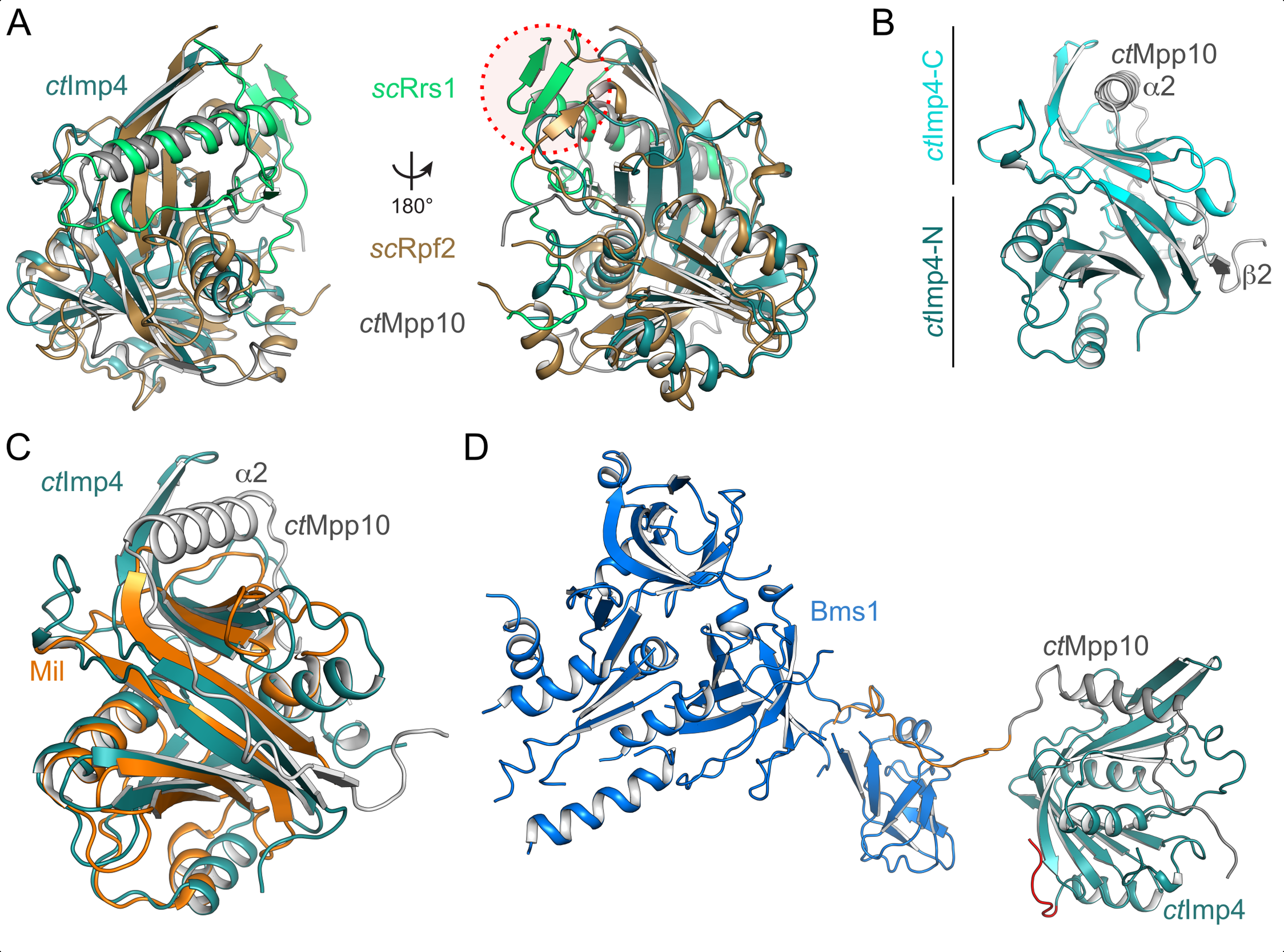

Supplement: S3 Fig — (A) Comparison of ctImp4-ctMpp10 with scRpf2-scRrs1 (PDB-ID: 5A53 [32]). The BRIX fold is conserved between ctImp4 (teal) and scRpf2 (light-brown). The BRIX-ligands, ctMpp10 (grey) and scRrs1 (lime-green) occupy the same position via a single α-helix. No β-augmentation is observed for ctMpp10 (red circle). (B) Structure of ctImp4 can be divided into sub-domains, the N- (teal) and C-terminal half (cyan). (C) Comparison of ctImp4 (teal) and the archeal Imp4-like protein Mil (orange). While ctImp4 is completed by ctMpp10 helix α2, no such ligand is known for Mil. (D) Modeling of the ctMpp10 N-terminus (orange) based on the yeast 90S cryo-EM density suggests an interaction with Bms1 (blue). (TIF) [file pone.0183272.s003.tif]
