# Supplementary material for: Mpp10 represents a platform for the interaction of multiple factors within the 90S pre-ribosome
Source: PLoS One. 2017 Aug 16;12(8):e0183272. doi: 10.1371/journal.pone.0183272 (PMC5558966; doi:10.1371/journal.pone.0183272)
Supplement: S1 Table — (PDF) [file pone.0183272.s004.pdf]

Table S1. Plasmids used in this Study

Plasmid	Relevant information	Source
pVA3-1	2μ, TRP1, GAL4 BD-murine p53 (72-390aa), pGBT9	Takara Bio Inc.
pTD1-1	2μ, LEU2, GAL4 AD-SV40 large T-antigen (84-708aa), pACT2	Takara Bio Inc.
pFA6A-natNT2	for genomic deletion disruption	Janke et al., 2004
pFA6a-FTpA-natNT2	Flag-TEV-ProtA,TADH1, for genomic C-terminal tagging	Thoms et al., 2015
pFA6a-3HA-Trp1	HA,TADH1, for genomic C-terminal tagging	Longtine et al., 1998
pGADT7-*ct*Mpp10	CEN, LEU2, PADH1, TADH1, N-terminal G4AD	Baβler et al., 2017
pGADT7-*ct*Imp4	CEN, LEU2, PADH1, TADH1, N-terminal G4AD	Baβler et al., 2017
pGADT7-*ct*Imp3	CEN, LEU2, PADH1, TADH1, N-terminal G4AD	Baβler et al., 2017
pGADT7-*ct*Utp3	CEN, LEU2, PADH1, TADH1, N-terminal G4AD	Baβler et al., 2017
pGADT7-*ct*Rps5	CEN, LEU2, PADH1, TADH1, N-terminal G4AD	This study
pGBKT7- *ct*Mpp10	CEN, TRP1, PADH1, TADH1, N-terminal G4BD	Baβler et al., 2017
pGBKT7- *ct*Imp4	CEN, TRP1, PADH1, TADH1, N-terminal G4BD	Baβler et al., 2017
pGBKT7- *ct*Imp3	CEN, TRP1, PADH1, TADH1, N-terminal G4BD	Baβler et al., 2017
pGBKT7- *ct*Utp3	CEN, TRP1, PADH1, TADH1, N-terminal G4BD	Baβler et al., 2017
pGBKT7- *ct*Rps5	CEN, TRP1, PADH1, TADH1, N-terminal G4BD	This study
YEplac181-P2 *ct*Rps5 p.Gal1-10-P1-pA TEV-*ct*Mpp10	2μ, LEU, for GAL co-expression in yeast, PGAL1-10, TADH1	This study
YEplac112-P2 *ct*Imp4 p.Gal1-10-P1-FLAG-*ct*Imp3	2μ, TRP1, for GAL co-expression in yeast, PGAL1-10, TADH1	This study
YEplac195-P2 empty p.Gal1-10-P1-2HA-*ct*Utp3	2μ, URA, for GAL co-expression in yeast, PGAL1-10, TADH1	This study
YEplac195-P2 empty p.Gal1-10-P1-*ct*Utp3	2μ, URA, for GAL co-expression in yeast, PGAL1-10, TADH1	This study
pGADT7-*ct*Mpp10 (1-162)	CEN, LEU2, PADH1, TADH1, N-terminal G4AD	This study
pGADT7-*ct*Mpp10 (184-467)	CEN, LEU2, PADH1, TADH1, N-terminal G4AD	This study
pGADT7-*ct*Mpp10 (468-601)	CEN, LEU2, PADH1, TADH1, N-terminal G4AD	This study
pGADT7-*ct*Mpp10 (602-785)	CEN, LEU2, PADH1, TADH1, N-terminal G4AD	This study
pET24d-GST-TEV-*ct*Mpp10 (59-90)	Kan^r , T7 promoter, lac operator	This study
pET24d-GST-TEV-*ct*Mpp10 (125-157)	Kan^r , T7 promoter, lac operator	This study

pET24d-GST-TEV	Kan^r , T7 promoter, lac operator	Thoms et al, 2015
pET15b-His-*ct*Utp3	Amp^r , T7 promoter, lac operator	This study
YEplac181-P2 *ct*Rps5 p.Gal1-10-P1- pA TEV-*ct*Mpp10 Δ59-90	2µ, LEU, for GAL co-expression in yeast, PGAL1-10, TADH1	This study
YEplac181-P2 *ct*Rps5 p.Gal1-10-P1- pA TEV-*ct*Mpp10 Δ125-157	2µ, LEU, for GAL co-expression in yeast, PGAL1-10, TADH1	This study
YEplac181-P2 *ct*Rps5 p.Gal1-10-P1- pA TEV-*ct*Mpp10 Δ59-157	2µ, LEU, for GAL co-expression in yeast, PGAL1-10, TADH1	This study
pet15b-His-*ct*Rps5	Amp^r , T7 promoter, lac operator	This study
pET24d-GST-TEV-*ct*Mpp10 (283-332)	Kan^r , T7 promoter, lac operator	This study
pET24d-GST-TEV-*ct*Mpp10 (283-410)	Kan^r , T7 promoter, lac operator	This study
YEplac181-P2 *ct*Rps5 p.Gal1-10-P1- pA TEV-*ct*Mpp10 Δ283-330	2µ, LEU, for GAL co-expression in yeast, PGAL1-10, TADH1	This study
p416 pTEF *sc*Mpp10	CEN, URA, PTEF, TCYC1	This study
p413 pTEF	CEN, HIS, PTEF, TCYC1	ATCC® 87362™
p413 pTEF *sc*Mpp10	CEN, HIS, PTEF, TCYC1	This study
p413 pTEF *sc*Mpp10 96-593	CEN, HIS, PTEF, TCYC1	This study
p413 pTEF *sc*Mpp10 Δ178-248	CEN, HIS, PTEF, TCYC1	This study
p413 pTEF *Sc*Mpp10 Δ299-375	CEN, HIS, PTEF, TCYC1	This study
p413 pTEF *sc*Mpp10 Δ428-448	CEN, HIS, PTEF, TCYC1	This study
p413 pTEF *sc*Mpp10 Δ96-287	CEN, HIS, PTEF, TCYC1	This study
