# Supplementary material for: Mpp10 represents a platform for the interaction of multiple factors within the 90S pre-ribosome
Source: PLoS One. 2017 Aug 16;12(8):e0183272. doi: 10.1371/journal.pone.0183272 (PMC5558966; doi:10.1371/journal.pone.0183272)
Supplement: S2 Table — (PDF) [file pone.0183272.s005.pdf]

Table S2. Yeast strains used in this study

Plasmid	Relevant information	Source
W303	*wild type*	Thomas and Rothstein, 1989
Imp4-FTpA	*W303, Matα, IMP4-FLAG-TEV-ProteinA::natNT2*	This study
PJ69-4A	*MATα, trp1-901, leu2-3,112, ura3-52, his3-200, gal4Δ, gal80Δ, LYS2::GAL1-HIS3, GAL2-ADE2, met2::GAL7-lacZ*	James et al., 1996
Mpp10 shuffle strain	*W303, Matα, mpp10::His3MX6, p416 pTEF -MPP10*	This study
