# Supplementary material for: Mpp10 represents a platform for the interaction of multiple factors within the 90S pre-ribosome
Source: PLoS One. 2017 Aug 16;12(8):e0183272. doi: 10.1371/journal.pone.0183272 (PMC5558966; doi:10.1371/journal.pone.0183272)
Supplement: S3 Table — (PDF) [file pone.0183272.s006.pdf]

Table S3: Data collection and refinement statistics.

	***ct*Imp4-*ct*Mpp10**
**Data collection**	
Wavelength (Å)	0.87260
Space group	$P6_4$
Cell dimensions	
a, b, c (Å)	112.88, 112.88, 44.00
α, β, γ (°)	90, 90, 120
Resolution (Å)	48.88-1.88 (1.95-1.88)*
R_{pim}	0.052 (0.557)
$I/s(I)$	9.15 (1.47)
CC1/2	0.997 (0.47)
Reflections total	181956 (17813)
Reflections unique	26155 (2516)
Completeness (%)	99.68 (96.88)
Multiplicity	7.0 (6.9)
**Refinement**	
R_{work}	0.1836 (0.3101)
R_{free}	0.2192 (0.3501)
No. atoms	
Protein	2252
Water	136
Ligands	4
B-factors	
Protein	34.81
Water	40.36
Ligands	47.38
R.m.s deviations	
Bond lengths (Å)	0.009
Bond angles (º)	0.95
Ramachandran plot	
Most favoured (%)	97.09
Disallowed (%)	0.00

* Values in parenthesis refer to the highest resolution shell.
